# The effectiveness of hyposaline treatments against host-attached salmon lice

**DOI:** 10.1038/s41598-019-43533-8

**Published:** 2019-05-06

**Authors:** Michael Sievers, Frode Oppedal, Ellen Ditria, Daniel W. Wright

**Affiliations:** 1 Institute of Marine Research, 5984 Matredal, Norway; 2 Australian Rivers Institute – Coast and Estuaries, Griffith University, Gold Coast, 4222 Queensland Australia; 3 Present Address: Australian Rivers Institute – Coast and Estuaries, Griffith University, Gold Coast, 4222 Queensland Australia

**Keywords:** Animal physiology, Ichthyology

## Abstract

Understanding how salinity affects marine parasites is vital to understanding their ecology and treatment, particularly for host-parasite systems that traverse marine and freshwater realms such as the globally important Atlantic salmon (*Salmo salar*), salmon louse (*Lepeophtheirus salmonis*) system. Growing concerns for wild fish populations, and decreased efficiencies and burgeoning costs of lice treatments for farmed fish has necessitated more environmentally and socially acceptable delousing procedures, such as hyposaline treatments. The effect of brackish water on *L*. *salmonis* following primary attachment is largely unknown, with experimental evidence derived mostly from unattached or newly attached copepodids, or adult stages. We aimed to understand how attached lice respond to hyposaline environments to assess effectiveness as a parasite management strategy and to help better define delousing areas used by wild fish. Louse development at 4, 12, 19 and 26 ppt, and survival at 4 ppt, decreased as exposure times increased, but survival was otherwise unaffected. Subjecting salmon to fluctuating, repeat exposures did not influence efficacy. We confirm that free-swimming stages are susceptible, and show that attached copepodids were more tolerant than previously predicted based on experiments on alternate development stages. These results improve our understanding of the utility of hyposaline treatments in aquaculture and self-treating in wild fish, and could apply to other fish-lice parasite systems. Further, these data are important for models predicting host-parasite interactions and can contribute to predictive models on the transmission dynamics of sea lice from farm to wild fish.

## Introduction

*Lepeophtheirus salmonis* (Krøyer, 1837; Copepoda, Caligidae) are crustacean copepods that feed on the blood, skin and mucus of their fish hosts, and are the primary ectoparasite of wild and farmed salmonids in the northern hemisphere^1,2^. Their life cycle comprises two nauplii stages, an infective copepodid stage that actively seeks a host, the immobile chalimus stages, the mobile pre-adult stages, and the reproductive adult stage^3,4^. *Lepeophtheirus salmonis* is the most prevalent pathogenic marine parasite affecting farmed Atlantic salmon and is regarded as one of the most significant threats to sustainable development of salmon culture^5,6^.

Infections irritate fish skin, reduce host growth rates, cause stress-induced mortality^7–9^, and ultimately reduce production efficiencies in cultured settings^10,11^. Wild fish – particularly juveniles^12^ – are similarly affected, and spillover of larval lice from farmed hosts has been directly linked to population declines of wild salmon^13–15^. Lice impose a substantial economic cost to the aquaculture industry through management actions and treatment regimes^16^. Previous attempts to prevent and treat sea lice in salmon aquaculture were largely reliant on the use of chemotherapeutants^15,17,18^. These treatments were expensive, caused high fish mortality^16,19,20^, and accumulated in the environment with flow-on effects for non-target organisms^21^. Sea louse control has more recently shifted towards thermal and mechanical delousing^22–24^, with similar welfare issues. These issues have spurred the continuing development and implementation of more environmentally sustainable and socially acceptable delousing procedures^25,26^.

Evidence from infested wild populations may help guide these ‘natural’ treatment alternatives. Copepodids of *L*. *salmonis* are stenohaline^27^, and infestation rates are lower on wild fish collected from areas with the lowest surface salinity^28^. Although in many circumstances wild fish may not be exposed to salinities capable of affecting louse abundance until they reach full freshwater^2,29^, wild sea trout with high *L*. *salmonis* loads do exhibit strong preferences for areas of lower salinity which suppresses louse infestation^30^. Furthermore, infested fish have been observed returning to brackish and freshwater prematurely, possibly as a result of heavy infestation, despite notable consequences for future fitness such as reduced growth and reproductive potential^31–33^.

Farmed salmon also exhibit reduced louse loads in low-salinity areas^28,34–36^ and experimental research shows that *L*. *salmonis* exhibit optimal survival and development at salinities closest to full seawater^37,38^. While full freshwater causes considerable mortality across stages^39–41^, early louse stages are generally more susceptible to hyposaline water than mature stages^37,38,41–43^. For example, copepodids at 1-dpi (days post-infection) are eliminated from salmon after exposure to freshwater (<1 ppt) for 1–3 h^41^, and a 3 h exposure at 4 ppt stopped copepodids establishing on salmon hosts in treatments where hyposaline exposure occurred immediately prior to or following infection events^37^, before primary attachment *via* second antennae occured^44^. Taken together, the application of hyposaline treatments within well- or snorkel-cages (i.e. semi-closed containment) may prove to be a useful treatment against lice in cultured settings.

The effect of brackish waters on *L*. *salmonis* survival and development has mostly been tested on unattached or newly attached copepodids, or adult stages. We aimed to better understand how *L*. *salmonis* copepodids post-primary attachment (i.e. 1-dpi) respond to hyposaline treatments to assess its effectiveness as a parasite management strategy within aquaculture, and to help better define delousing areas used by wild fish.

## Materials and Methods

### Louse collection and cultivation

Egg strings used to initiate a culture stock at the Institute of Marine Research in Matre, Norway were collected in October 2017 from the experimental salmon farms at Solheim and Smordalen, in Masfjorden, on the southwest coast of Norway. For our experiments, adult female *L*. *salmonis* with egg strings were continually collected from previously infected Atlantic salmon following sedation (metomidate 10 mg L^−1^) throughout April–May 2018. Egg strings and planktonic larval stages (nauplius and copepodids) were incubated at 15 °C in flow-through (~1 L/min, 34 ppt salinity) plastic containers (20 × 15 × 12 cm; L × W × H) with 150 μm mesh bases^45^.

### Survival and development of 1-dpi copepodids

#### Infection protocol

Active copepodids were collected from incubators by sieving water through 150 μm mesh and rinsing with fresh seawater into a 2 L beaker. Copepodid density was assessed by mixing the sample and removing 10 ml aliquots into a counting chamber. The number of active copepodids per aliquot was enumerated in quintuplicate and the total number of copepodids in the beaker estimated. The entire sample was thoroughly mixed and decanted into four separate 500 mL beakers.

For each trial week, Atlantic salmon post-smolts of farmed origin were collected from a single, onshore holding tank containing approximately 1500 fish, and were held in four 400 L holding tanks (93 × 93 × 55 cm; L × W × W) at full salinity (34 ppt) and 15 °C under a flow rate of 20 L/min (32–33 fish per tank for single and 26–27 for repeat exposures; Supplementary Fig. 1). No fish were infested with lice whilst in this onshore holding tank. Fish were not measured prior to experimentation to minimise handling and stress. Fish were infected by adding copepodids (~2 days post-moult; 1971 ± 303; mean ± SD) across the four tanks after stopping the incoming flow and reducing the water level to 20 cm to increase encounter rates (Supplementary Fig. 1). Lice were allowed to attach under no flow for 20 min with aeration, during which time, fish were scared every 5 min by waving above the tank to increase host-parasite interaction. After the 20 min, flow was reintroduced slowly at 2 L/min for an additional 40 min to refill the tank, after which, flow was returned to 20 L/min and flow-through reinstated.

#### Salinity levels and exposure durations

We tested five exposure durations (0 (control), 3, 9, 24 and 72 h) for four salinity levels (4, 12, 19 and 26 ppt). These salinities match those used in a previous trial with *L*. *salmonis* in Scotland^37^. We chose to conduct experiments over a 72 h period as an attempt to completely cover the period that lice would be at the copepodid stage based on known temperature-dependent developmental rates^46^. The experimental facility, with groups of multiple tanks fed from the same header tank, precluded a completely randomised design. Instead, we ran one salinity treatment at a time, looking at the various durations of exposure (randomly assigned) to a different salinity treatment each week. Therefore, initial infection levels differed slightly for each salinity level due to variable numbers of egg strings produced each week, and because precise enumeration of copepodid abundance is impractical. Water temperature (15 °C) and salinity levels within tanks were fully automated and tested using custom made computer software (SD Matre, Normatic AS, Nordfjordeid, Norway), and appropriate salinity levels were set the day prior to exposure. As part of the same experiment, we also conducted repeated brackish water exposures at 4, 12 and 19 ppt (one salinity per trial), where we exposed fish for 1 h once, twice or thrice per day (with 1 h full seawater between, and full seawater for the remainder of the 24 h) for three consecutive days, with controls of no exposure duration (i.e. full seawater for 24 h).

#### Post-infection exposure to brackish water

**Figure 1:**
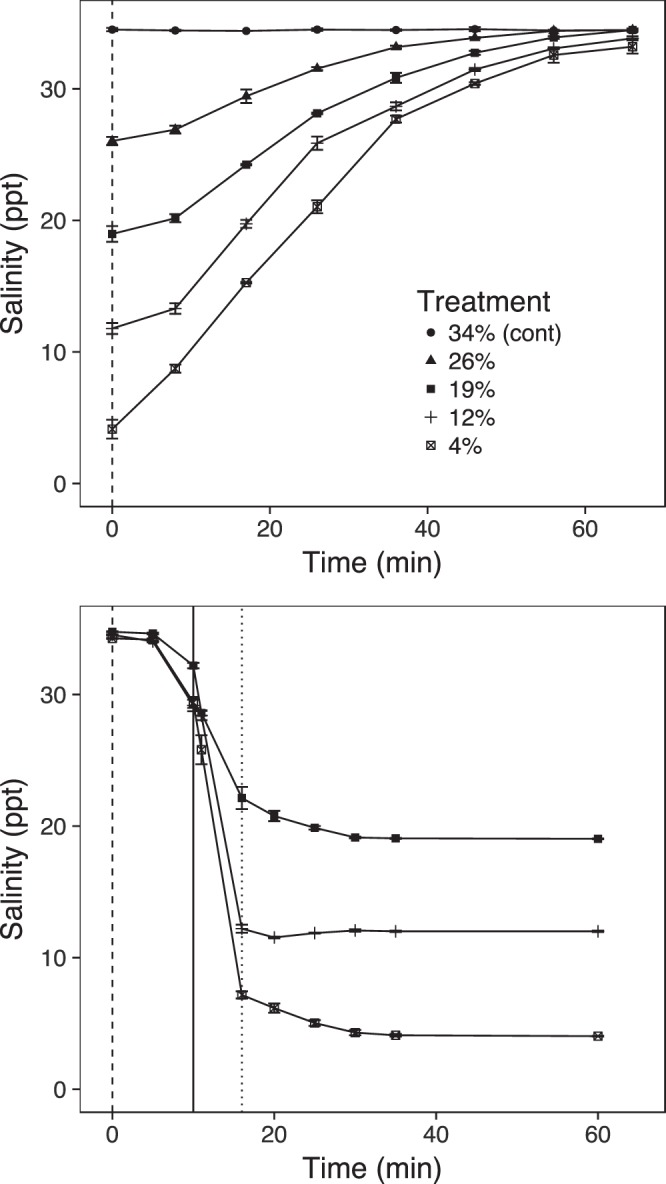
Example of change in salinity profile over time after tank inflow had been switched to full (34 ppt) seawater. This figure shows the results of a trial run. Given flow rate and temperature does not change across treatment exposures, rates of salinity change back to full seawater should be identical regardless of exposure duration. Dashed lines represent the time when header tanks were switched to the required salinity, the solid line represents when tank water level was dropped immediately before freshwater was pumped in, and the dotted line represents when pumping ceased. Points and error bars are means with standard errors, with each n = 3.

Fifteen treatment tanks with the same dimensions, temperature and flow rate as the infection tanks were used for trials each week (n = 3 tanks per duration). At 1-dpi, two fish from each of the four infection tanks were randomly added to each of the treatment tanks containing water at the appropriate salinity, resulting in eight fish per tank (Supplementary Fig. 1). After each respective exposure duration, incoming flow was switched to full seawater (34 ppt). Therefore, fish were exposed to a rapid decrease in salinity, and then a more gradual return to full seawater (Fig. 1).

**Figure 2:**
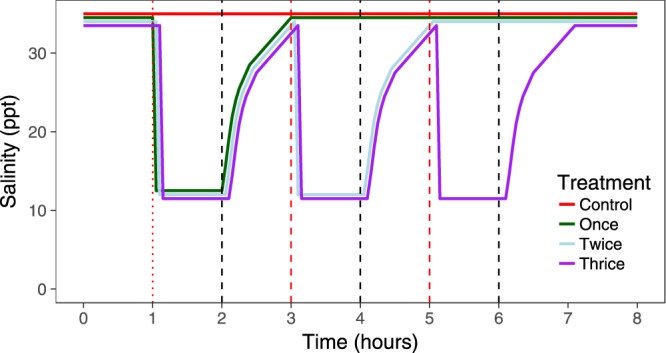
Schematic of salinity changes in the repeat exposures based on 12 ppt. Salinity changes occurred for three consecutive days. Lines are staggered to aid visualisation. Red vertical dashed lines indicate header tanks being set to 12 ppt and tanks being flushed, and black vertical dashed lines indicate header tanks being set to full (34 ppt) seawater. The dotted red line is the initial fish transfer on day one (i.e. immediately into brackish water), and a flush for days two and three.

Twelve treatment tanks were used for the repeated exposure trials (n = 3 tanks per frequency). Eight fish were distributed into each tank and underwent the first exposure to brackish water (i.e. immediately) as above. After 1 h, header tanks were set to full seawater (i.e. 34 ppt). After an additional hour, we set header tanks to the required salinity (for those tanks requiring a second exposure) and dropped the tank water level in all tanks to 10, 20 or 30 cm for the 4, 12 and 19 ppt treatments, respectively (Fig. 2). We then pumped either freshwater (1 ppt) or seawater (34 ppt) dependent on the treatment required from storage tanks with a 120 L/min pump (Fig. 1). This was repeated for a third exposure. This way, all tanks underwent the lowering of water level and rapid filling with pumped water (i.e. controlling the procedure). This was repeated for three consecutive days (Fig. 2).

Apart from during treatment with brackish water, all tanks were maintained at full seawater. At all times, tanks were on a 12:12 h light:dark cycle, and fish were fed in excess using automated feeders throughout the duration of the experiment.

#### Infection assessment

We sedated fish with metomidate (10 mg L^−1^) and lethally sampled all eight fish from each tank at 6-dpi to assess infection levels and fish welfare. We counted and staged lice, and recorded the length, mass and salmon welfare index model score quantifying the condition of the skin, fins, snout and eyes of each fish^47^.

### Survival of free-swimming copepodids

During experiments on 1-dpi copepodids, we also assessed free-swimming copepodid survival by closely replicating the experimental design of Bricknell and colleagues^48^. Copepodids were collected from the same incubators used for the above experiments, passed through a 150 μm sieve, transferred to a 2 L beaker with seawater, and left to stand for 5 min below a 60 W light source. Actively swimming copepodids were distributed (7.2 ± 2.8; mean ± SD) into individual 100 mL beakers held in a 15 °C waterbath. The allocation of copepodids across salinities occurred in a staggered process, with each of the six successive beakers for each salinity (see below) filled with copepodids every 2 min. We used a total of 54 beakers containing water at 1, 4, 9, 12, 16, 19, 23, 26, 34 ppt (six per salinity; measured using WTW conductivity meter 315i, Xylem Analytics, Germany). This water is a mixture of full saline water from 90 m depth in Masfjorden and freshwater supply from local rivers, and is filtered and UVC treated. At each of 1, 2, 3, 4, 5, and 6 h post exposure to the given salinity, the contents of a single beaker were poured through a 150 μm sieve, and the copepodids placed into a petri dish with full seawater. After an additional hour, we scored copepodids as being active (swimming or responsive to prodding) or dead. Full seawater was used within the petri dish and an hour was allowed for recovery as some lice remain completely unresponsive and appear dead in lowered salinities but recover when returned to full seawater (E. Ditria, *unpublished data*). The experiment was repeated five times, yielding five independent replicates for each combination of salinity (1–34 ppt) and exposure duration (1–6 h). The staggered nature of the initial distribution allowed each beaker to be assessed exactly every hour.

### Statistical analysis

Given that salinity levels were tested in different weeks, and thus with different broods of copepodids, we analysed each trial separately (precluding the addition of a tank random effect). Louse abundance was converted to a density (lice/cm^2^ of fish surface area) using the formula from Frederick and collegues^49^ which accounts for the correlation between host size and parasite load. We averaged lice numbers for each tank to avoid pseudoreplication. We fitted linear models to the louse density on fish at the end of experiments, including duration (single exposures) or exposure type (repeat exposures) as fixed effects. Total fish per salinity was 120 (n = 15 replicates) for single exposures and 96 (n = 12 replicates) for repeat exposures. Because we did not compare across salinities (and thus with individual tanks used more than once), tank was not included as a random effect. For single exposures, we conducted post-hoc Tukey’s tests comparing durations using adjusted alpha values to reduce the type I error rate. To investigate developmental rates, we conducted non-parametric Kruskal-Wallis tests with duration (single exposures) or exposure type (repeat exposures) fitted as fixed effects on the proportion of lice that had developed beyond the chalimus II stage (99% of all lice were either chalimus I or chalimus II). When significant, we performed pairwise comparisons using Dunn’s tests. Fish SWIM scores were not analysed statistically as fish within each trial were in very similar condition, so variances were often zero and mean differences negligible (Supplementary Table 1).

To assess survival of free-swimming copepodids, we fitted a generalised linear model to the proportion of copepodids that were alive in each beaker, with salinity and hour fitted as fixed effects, experimental day as a random effect, and used a quasibinomial distribution. We estimated LT_50_ values (time at which 50% of the population is dead) from the fitted model predictions of survival over time and estimated confidence intervals using the delta method in the R package emdbook^50^. We set mortality to 100% at 12 days for all salinities (mortality of free-swimming copepodids will be 100% after 12 days^1^) so that survival curves have realistic end-points.

We assessed normality and homogeneity of variances before all parametric analyses using Q-Q and Levene’s tests, respectively. Transformations (specified in tables and figures) were performed to meet statistical assumptions when appropriate. We performed analyses in R 3.2.2^51^.

The work was conducted in accordance with the laws and regulations controlling experiments and procedures on live animals in Norway following the Norwegian Regulation on Animal Experimentation 1996 (Ethics approval 12935 and 14133), and was approved by the institutional Committee at the Norwegian food authorities (Mattilsynet).

## Results

### Survival and development of 1-dpi copepodids

**Figure 3:**
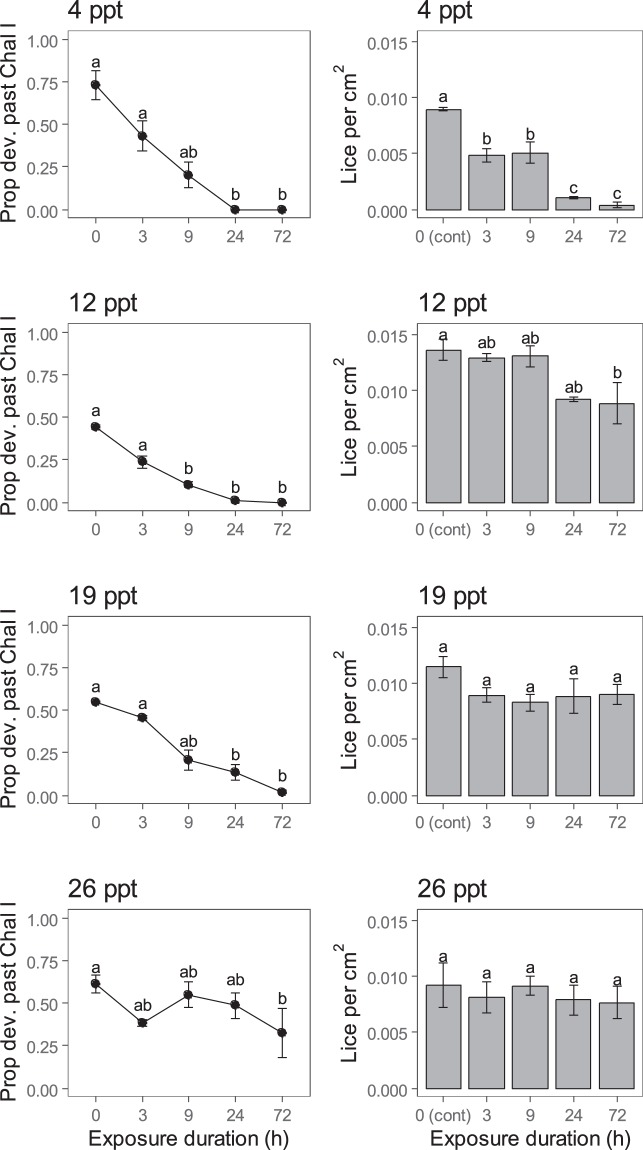
Density and development (proportion of surviving lice (*Lepeophtheirus salmonis*) that developed past chalimus I stage) of lice following single exposure to brackish water at 4, 12, 19 and 26 ppt for 3, 9, 24 and 72 hours. Different letters indicate statistically significant differences from Tukey’s tests with adjusted-p. Columns and points represent means, and error bars are standard errors, with each n = 3.

For the single exposure treatments, fish at the time of sampling were 31.8 ± 0.1 cm (Mean ± SE hereafter) in length and 315.6 ± 4.5 g in weight, and showed no signs of morphological issues (Supplementary Table 1). Assuming equal initial infection levels across the four infection tanks, duration of exposure to brackish water at 4 and 12 ppt influenced the density of lice attached to fish, but this was not the case at 19 and 26 ppt (Table 1; Fig. 3). With exposure to 4 ppt for 3, 9, 24, the proportion of lice that were alive decreased to 54, 56, 12 and 4%, respectively, relative to controls (Fig. 3). At 12 ppt, survival was only significantly reduced after 72 h exposure, although louse levels after 24 h were similar to those after 72 h (Fig. 3).

**Table 1:** Output from one-way ANOVA for 1-dpi lice (Lepeophtheirus salmonis) survival following single exposure to 4, 12, 19, or 26 ppt for 3, 9, 24 or 72 hours, and repeated exposures over three days to 4, 12 or 19 ppt for one hour once, twice or thrice per day.

	Salinity	Factor	df	MS	F	p
*Single exposure*	4 ppt	**Duration**	**4**	**0**.**0006**	**45**.**54**	<**0**.**001**
		Error	10	0.0000		
	12 ppt	**Duration**	**4**	**0**.**0002**	**4**.**74**	**0**.**02**
		Error	10	0.0001		
	19 ppt	Duration	4	0.0001	1.44	0.29
		Error	10	0.0000		
	26 ppt	Duration	4	0.0000	0.25	0.90
		Error	10	0.0001		
*Repeat exposure*	4 ppt	**Frequency**	**3**	**0**.**0002**	**8**.**68**	**0**.**007**
		Error	8	0.00002		
	12 ppt	Frequency	3	0.00002	1.65	0.25
		Error	8	0.00001		
	19 ppt	Frequency	3	0.00001	0.88	0.49
		Error	8	0.00001		

Boldface values are statistically significant at alpha = 0.05.

Exposure to brackish water slowed louse development at 4 ppt (Kruskal-Wallis ${\chi }_{4}^{2}$ = 13.3, p = 0.01), 12 ppt (K-W ${\chi }_{4}^{2}$.  = 13.4, p = 0.01) and 19 ppt (K-W ${\chi }_{4}^{2}$ = 13.0, p = 0.01), but not at 26 ppt (K-W ${\chi }_{4}^{2}$ = 6.0, p = 0.20) although there was evidence that 72 h exposure to 26 ppt reduced development relative to controls (Fig. 3). While 44–73% of lice developed into chalimus II in the control groups, less than 2% had done so after 24 or 72 h exposure to 4 or 12 ppt, or 72 h to 19 ppt (Fig. 3).

**Figure 4:**
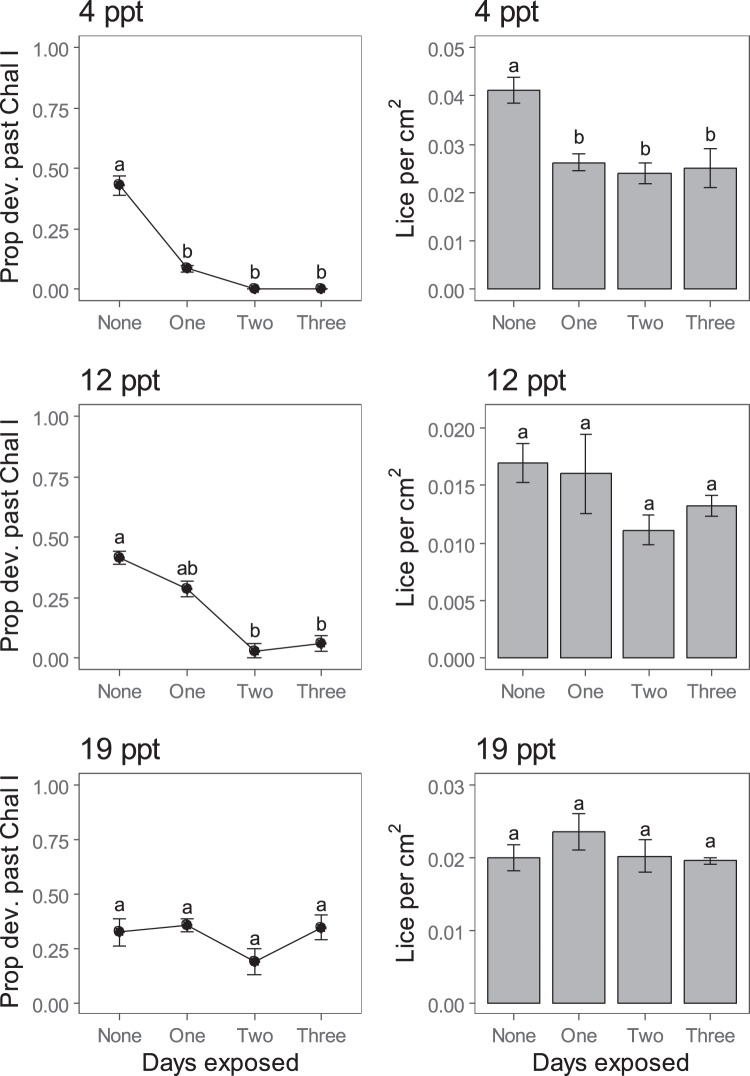
Density and development (proportion of surviving lice (*Lepeophtheirus salmonis*) that developed past chalimus I stage) of lice following repeated exposure to brackish water at 4, 12 and 19 ppt for 1 hour once, twice or thrice per day for three consecutive days. Different letters indicate statistically significant differences from Tukey’s tests with adjusted-p. Note the differing y-axis values. Columns and points represent means, and error bars are standard errors, with each n = 3.

For the repeat exposure treatments, fish at the time of sampling were 23.1 ± 0.4 cm in length and 122.1 ± 6.2 g in weight and were in good condition (Supplementary Table 1). Exposure to brackish water at 4 ppt reduced the density of lice attached to fish, but the number of exposures per day had no affect (Fig. 4). No level of repeat exposure at 12 or 19 ppt was effective (Table 1; Fig. 4).

Repeated exposure to brackish water slowed louse development at 4 ppt (Kruskal-Wallis ${\chi }_{3}^{2}$ = 10.6, p = 0.01) and 12 ppt (K-W ${\chi }_{3}^{2}$ = 9.6, p = 0.02), but not at 19 ppt (K-W ${\chi }_{3}^{2}$ = 3.6, p = 0.31; Fig. 4). Again, there was no evidence that increasing the number of exposures per day affected development more strongly (Fig. 4).

### Survival of free-swimming copepodids

Survival of free-swimming copepodids decreased as salinity decreased and exposure time increased (Table 2; Supplementary Fig. 2). Model estimates indicate that at 12 ppt and below, more than half the lice will be dead in less than 3 hours (Table 2). Model estimates and their variances for the highest salinities (e.g. 26 and 34 ppt) should be interpreted with caution due to high survival over the 6 h experimental period necessitating considerable extrapolation.

**Table 2:** LT_50_ values (time in hours at which 50% of the *Lepeophtheirus salmonis* population will be dead) and ± SE based on modelled parameter estimates from a generalized linear model, family quasibinomial with experimental day a random effect.

Salinity	LT_50_	
	*Bricknell*	*This study*
1	NA	0.4 (±0.20)
4	NA	0.9 (±0.20)
5	<1	NA
9	<1	2.0 (±0.15)
12	<1	2.9 (±0.15)
16	4	4.5 (±0.25)
19	6	6.3 (±0.35)
23	8	9.9 (±0.65)
26	11	14.6 (±1.10)
29	14	NA
33	22	NA
34 (control)	NA	128.6 (±7391)
36	25	NA

Very high survival over 6 h in the control group led to extrapolation and very large standard deviation. Bricknell refers to data obtained in similar experiments by Bricknell and colleagues^48^.

## Discussion and Conclusions

An essential step in the effective management of *L*. *salmonis* for both wild and cultured fish is to understand how changes in environmental conditions affect lice. We show that the survival of 1-dpi attached lice was only affected at very low salinities or over long exposure durations at moderately low salinities. This contrasts with expectations that attached copepodids would be highly susceptible due to a lack of osmoregulation. On the other hand, mortality of free-swimming lice, as expected, occurred at much higher salinities after short exposure durations. For example, we found no evidence that exposure to salinities of 19 ppt killed attached lice, even after 72 h, but the LT_50_ – the time taken to kill half the free-swimming copepodids – for 19 ppt was only 6.3 hours. Repeated exposures involving multiple abrupt changes in salinity did not decrease survival any more than single treatments of the equivalent total exposure time.

The LT_50_ values estimated here confirm previous studies investigating the lethality of hyposaline water on free-swimming lice stages^42,52^, but were consistently higher than (although still comparable to) those from Bricknell and colleagues^37^. We suggest three potential reasons: (1) the copepodids we used may be inherently more tolerant to brackish water than those used by Bricknell (perhaps due to a greater scope for phenotypic plasticity), (2) lice may have evolved some resistance to brackish water since Bricknell (perhaps due to increased adoption of freshwater treatments in aquaculture), or (3) the shorter recovery time in Bricknell (10 min *verses* 1 h) may have underestimated survival rates. Our experimental design does not allow us to separate these possible mechanisms, nor was it our intention.

Our data show that allowing copepodids to complete primary attachment before hyposaline treatment greatly increases survival relative to the survival of free-swimming copepodids, and newly attached lice. It has been suggested that copepodids and chalimus stages cannot use ions obtained from the host to replace those lost to the hypoosmotic environment based off the research by Hahnenkamp and Fyhn^52^. Wright and colleagues^41^ suggested that although these pathways might be weak or non-existent for copepodids, they may be high functioning for chalimus and pre-adult stages. Early research found that copepodid energy stores stabilise following primary attachment at 1-dpi, suggesting that attached copepodids are actively feeding on hosts^53^. This may provide some osmoregulatory capacity to 1-dpi copepodids and allow greater survival and development under brackish conditions. Our results support this and suggest that copepodids may be accessing host-dependent osmoregulatory mechanisms and gain ions from feeding, but this remains a key area for future research, as other potential explanations exist, such as lice benefitting from fish mucous or feeding.

Since we were not able to examine different salinities within each successive trial (we compared durations), we did not analyse this experiment as a full factorial crossed design. In other words, comparisons of lice survival across salinities for each duration should be done so with caution. However, given we randomly selected fish throughout the experiment from a single population housed together, each successive trial was conducted immediately after the previous (i.e. all were conducted within a short time-frame), and all lice were from the same batch of parental lice, with any one batch containing copepodids from at least 20 different females of various age, we feel some qualitative comparison is justifiable.

Developmental rates in terms of the proportion of lice that had developed beyond chalimus I were also highly affected by exposure to brackish water. The biological and commercial significance of these reductions, however, are uncertain as we cannot estimate absolute reductions to development (i.e. how long development is reduced between stages). If development to breeding stages is substantially slowed by exposure to brackish water, re-infections occurring within farms could be reduced. However, our evidence may be biased by mortality, as we were unable to separate the effect of survival from developmental rates^54^. Future experiments should aim to document louse development at different salinities across more life stages by sampling subsets of fish (and thus lice) through time, and accounting for possible confounding with survival rates.

Sea lice are renowned for their capacity to evolve resistance to chemical and drug treatments^55,56^. It is also possible that louse populations are evolving tolerance to hyposaline water as *L*. *salmonis* populations show considerable family-level genetic variation, with links to temperature and salinity tolerance^57^. Given the potential for rapid evolution, any commercial treatments should aim to kill a very high proportion of the louse population, just as for any form of disease treatment. Proper implementation of louse control measures is critical to ensure continued efficacy of treatments for the protection of both cultured and wild fish. Our results suggest that many commercially achievable salinity levels (e.g. a minimum of 4–5 ppt throughout the upper 2 m for a well-cage; Daniel Wright *unpublished data*) are ineffective at killing all attached lice, and thus, may potentially drive resistance. However, the potential for resistance to evolve exists for essentially all treatment methods applied at commercial scales today and cannot be a reason for abandoning its use. Instead, we should incorporate hyposaline treatments within a cyclical treatment regime (as the important infective and newly attached stages are still susceptible), whereby different treatment types are applied in succession so that resistance to any one treatment is stymied. Teaching and encouraging natural behaviours (e.g. jumping, surface activity of hosts) during treatment would also enhance treatment efficacies^58^.

Finally, temperature has a strong influence on louse survival, attachment success and development^1^. Temperature can also influence the susceptibility of lice to salinity, for example, *Acartia tonsa* copepodids are more tolerant of brackish water at higher temperatures^59^. Our study was conducted at 15 °C, a relatively warm temperature for Norwegian salmon farms North of Rørvik (mid Norway), but not outside natural ranges in most salmon producing countries (e.g. min to max; approximately −1 °C to 22 °C^46^). Even still, the effect of brackish water for treating lice – for both wild and cultured fish – may be more effective at colder temperatures.

The use of brackish water and freshwater is a key defense that wild fish have against lice burdens, and our study provides important information on what exposures are required once lice complete primary attachment. Although some wild salmon may not be exposed to salinities capable of affecting lice for long enough^2,29^, this information can be incorporated into models predicting host-parasite interactions^34,60^ and contribute to predictive models on transmission dynamics of sea lice from farm to wild fish^61,62^. The details herein may also be broadly applicable to other fish host-parasite systems involving external ectoparasites, and we hope these insights spur research on similar parasites around the world such as *Caligus elongatus* and *C*. *rogercresseyi*^63,64^.

Ultimately, our results suggest that hyposaline treatments in cultured settings would ideally occur in full freshwater^41^, which raises concerns around some commercial applications given desalinated water becomes more saline when added to well- or snorkel-cages. Still, infective and newly attached stages are highly susceptible and hyposaline treatments offer a comparatively environmentally and welfare friendly method to reduce lice loads on farmed fish. Continued understanding of how larval lice respond to hyposaline environments can help further tailor parasite management strategies, reducing our reliance on chemicals, and help better define delousing areas used by wild salmonids. Future work should aim to investigate the capacity of copepodids to access host ions, test the effect of finer scale salinity gradients to attached copepodid survival and development, and examine the current rate, and future potential, of lice to evolve resistance to fresh and brackish water treatments.

## Supplementary information

Supplementary Information

## Data Availability

Upon acceptance, all data will be made publicly available on Fig. Share.
