# Supplementary material for: The effectiveness of hyposaline treatments against host-attached salmon lice
Source: Sci Rep. 2019 May 6;9:6976. doi: 10.1038/s41598-019-43533-8 (PMC6502938; doi:10.1038/s41598-019-43533-8)
Supplement: Supplementary file 1 — Supplementary Information [file 41598_2019_43533_MOESM1_ESM.pdf]

The effectiveness of hyposaline treatments against host-attached salmon lice

Supplementary Information

Michael Sievers[1, 2*], Frode Oppedal[1], Ellen Ditria[1, 2] and Daniel W Wright[1]

[1] Institute of Marine Research, Matredal, Norway, 5984

[2] Australian Rivers Institute, Global Wetlands Program, Griffith University, Gold Coast, Queensland, Australia

*Corresponding author: Michael Sievers (m.sievers@griffith.edu.au)

Current address: Australian Rivers Institute, Griffith University, Gold Coast, Queensland, Australia.

Telephone: +61 7 5678 0562

Running title: Host-attachment reduces susceptibility to brackish water

Supplementary figure captions

Supplementary Figure 1. Experimental design for single exposure treatments. Each blue square represents a 400L tank. See methods of description of design.

Supplementary Figure 2. Proportion survival of free-swimming copepodids (*Lepeophtheirus salmonis*) exposed to brackish water. Lines represent modelled parameter estimates from a generalized linear model, family quasibinomial to predict LT_{50} (time at which 50% of the population will be dead; Table 1), and 95% confidence intervals (delta method) are displayed along the horizontal line at y=0.50.

Supplementary Table 1. Mean ± SE (n = 3 tanks, 8 fish subsampled per tank) salmon welfare index model (SWIM) score values for fins, skin and eyes for Atlantic salmon (*Salmo salar*). For variable descriptions see Stien et al 2013. No significant differences amongst treatments were observed.

	Salinity	Exposure	Fin score	Skin score	Eye score
Single exposure	4 ppt	Control	2.00 ± 0.00	2.75 ± 0.14	1.21 ± 0.08
		3 h	2.00 ± 0.00	2.79 ± 0.18	1.21 ± 0.11
		9 h	2.00 ± 0.00	3.00 ± 0.14	1.25 ± 0.19
		24 h	2.00 ± 0.00	2.92 ± 0.11	1.25 ± 0.07
		72 h	1.92 ± 0.04	3.08 ± 0.04	1.08 ± 0.04
	12 ppt	Control	2.00 ± 0.07	3.00 ± 0.19	1.08 ± 0.04
		3 h	2.00 ± 0.00	3.42 ± 0.22	1.08 ± 0.08
		9 h	2.00 ± 0.00	3.17 ± 0.11	1.08 ± 0.08
		24 h	1.96 ± 0.08	2.79 ± 0.22	1.00 ± 0.00
		72 h	2.00 ± 0.00	2.75 ± 0.14	1.00 ± 0.00
	19 ppt	Control	2.04 ± 0.08	3.38 ± 0.14	1.13 ± 0.07
		3 h	2.04 ± 0.04	3.08 ± 0.11	1.04 ± 0.04
		9 h	1.83 ± 0.11	3.13 ± 0.07	1.13 ± 0.13
		24 h	1.92 ± 0.04	3.04 ± 0.08	1.00 ± 0.00
		72 h	2.00 ± 0.00	3.17 ± 0.08	1.00 ± 0.00
	26 ppt	Control	2.04 ± 0.04	2.42 ± 0.30	1.42 ± 0.23
		3 h	1.92 ± 0.08	2.92 ± 0.27	1.21 ± 0.11
		9 h	1.96 ± 0.04	2.46 ± 0.04	1.13 ± 0.13
		24 h	2.00 ± 0.00	2.67 ± 0.08	1.25 ± 0.00
		72 h	2.08 ± 0.04	3.08 ± 0.15	1.21 ± 0.08
Repeat exposure	4 ppt	Control	2.00 ± 0.00	3.42 ± 0.34	1.00 ± 0.00
		1 × 1 h × 3 d	2.00 ± 0.00	3.50 ± 0.38	1.00 ± 0.00
		2 × 1 h × 3 d	2.00 ± 0.00	3.29 ± 0.43	1.00 ± 0.00
		3 × 1 h × 3 d	1.96 ± 0.12	3.33 ± 0.47	1.00 ± 0.00
	12 ppt	Control	2.00 ± 0.00	3.42 ± 0.04	1.00 ± 0.00
		1 × 1 h × 3 d	2.00 ± 0.00	3.50 ± 0.07	1.00 ± 0.00
		2 × 1 h × 3 d	2.00 ± 0.00	3.29 ± 0.17	1.00 ± 0.00
		3 × 1 h × 3 d	2.00 ± 0.00	3.33 ± 0.27	1.00 ± 0.00
	19 ppt	Control	2.00 ± 0.00	3.63 ± 0.19	1.04 ± 0.04
		1 × 1 h × 3 d	2.00 ± 0.00	3.42 ± 0.25	1.00 ± 0.00
		2 × 1 h × 3 d	2.04 ± 0.04	3.29 ± 0.11	1.00 ± 0.00
		3 × 1 h × 3 d	2.04 ± 0.04	3.83 ± 0.25	1.08 ± 0.08

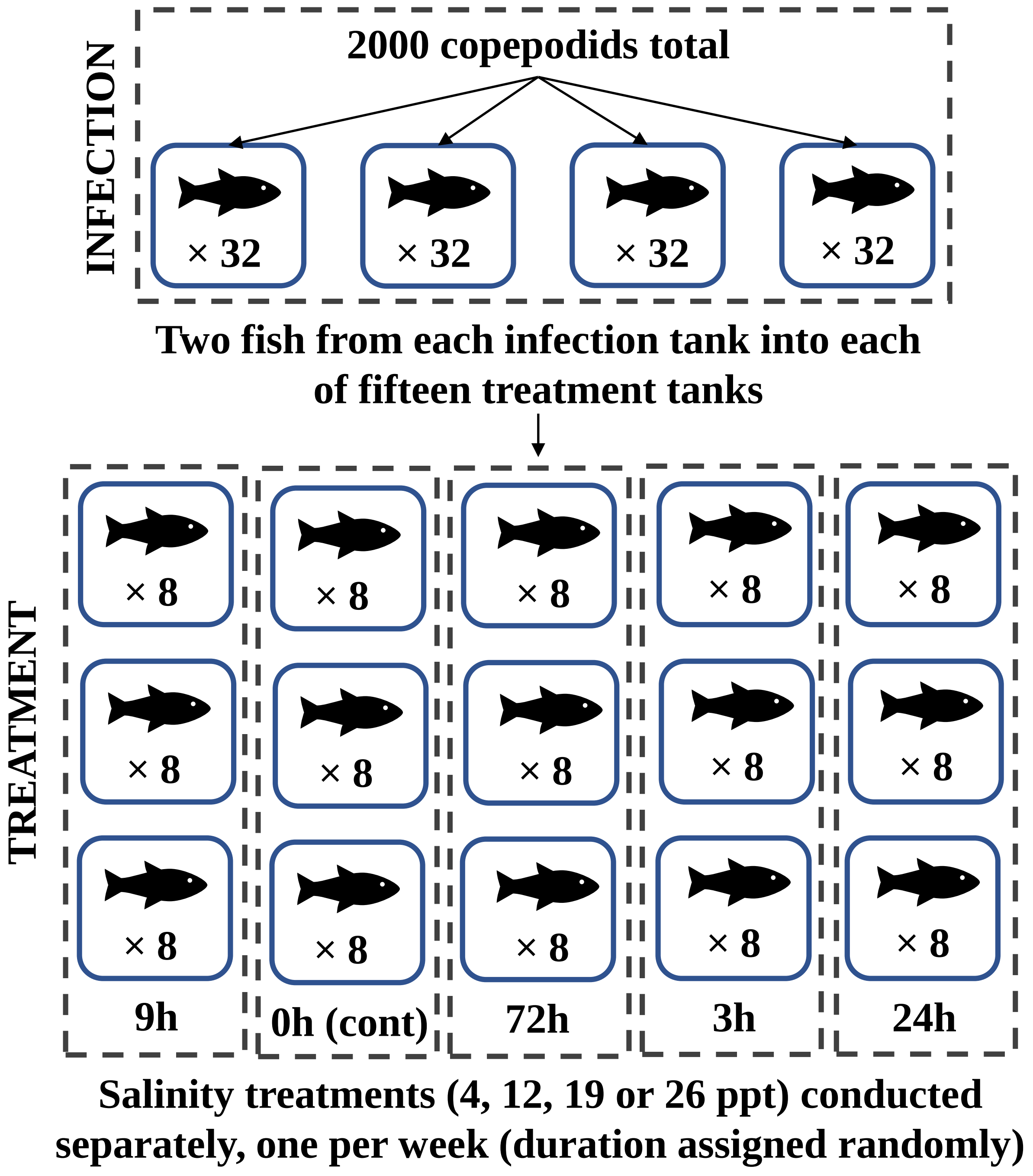
INFECTION
2000 copepodids total
× 32
× 32
× 32
× 32
Two fish from each infection tank into each of fifteen treatment tanks
TREATMENT
× 8
9h
0h (cont)
72h
3h
24h
Salinity treatments (4, 12, 19 or 26 ppt) conducted separately, one per week (duration assigned randomly)

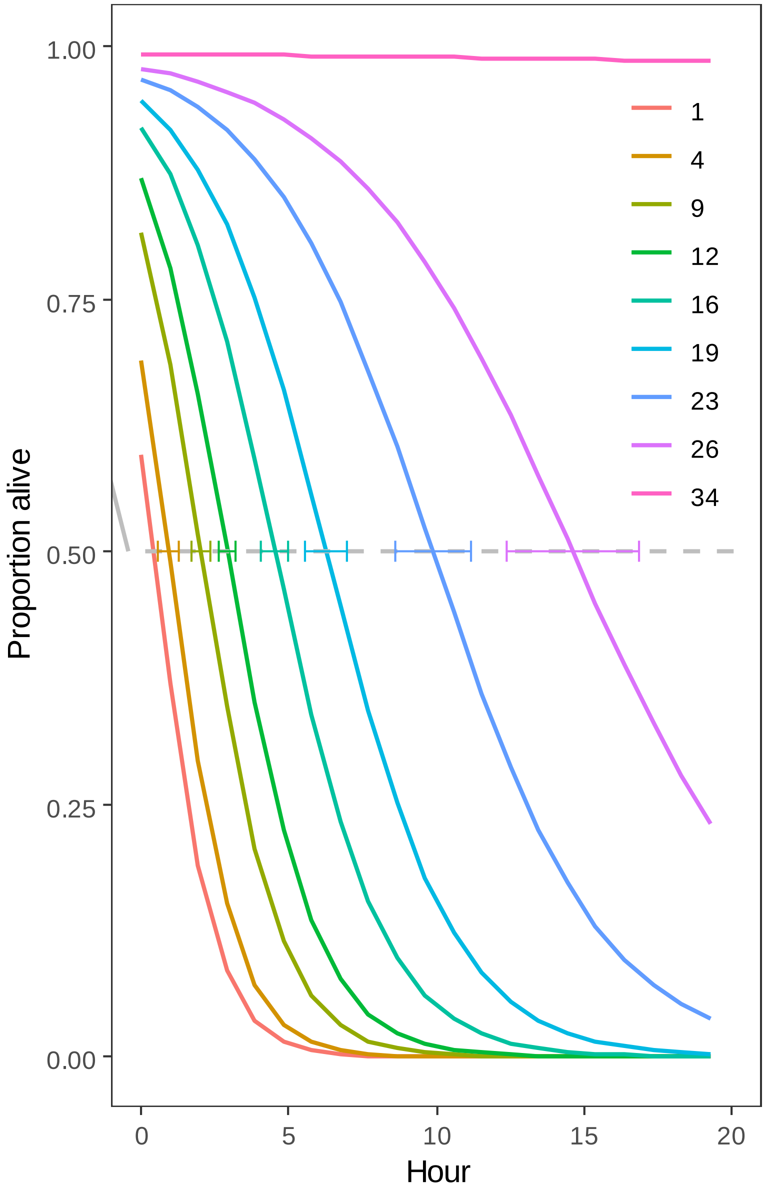

Proportion alive
1.00
0.75
0.50
0.25
0.00
0
5
10
15
20
Hour
1
4
9
12
16
19
23
26
34
